# Effects of Nitrogen and Shading on Root Morphologies, Nutrient Accumulation, and Photosynthetic Parameters in Different Rice Genotypes

**DOI:** 10.1038/srep32148

**Published:** 2016-08-25

**Authors:** Shenggang Pan, Haidong Liu, Zhaowen Mo, Bob Patterson, Meiyang Duan, Hua Tian, Shuijing Hu, Xiangru Tang

**Affiliations:** 1 College of Agriculture, South China Agricultural University, Guangzhou, China; 2 College of Agriculture and Life Science, North Carolina State University, NC, United States; 3 Department of Plant pathology, North Carolina State university, NC, United States

## Abstract

Nitrogen availability and illumination intensity are two key factors which affect rice growth. However, their influences on total nitrogen accumulation, photosynthetic rate, root morphologies, and yields are not fully understood. We conducted two field experiments to (1) evaluate the effects of shading under different N treatments on photosynthetic parameters, root morphologies, total nutrient accumulation, and grain yields of rice; and (2) elucidate the relationship between total nutrient accumulation and root morphologies under different shading conditions and nitrogen treatments. Three nitrogen rates, three shading treatments, and three different rice cultivars were used in two field experiments. Double shading during the grain-filling stage decreased total nutrient accumulation, altered root morphological characteristics, and decreased yields in rice. There were also significant interaction effects between nitrogen and shading on photosynthetic rate, transpiration rate, and total root length, root superficial area, and root volume. Significant interactions were found among cultivars and shading for photosynthetic rate and transpiration rate. Correlation analysis revealed that total nitrogen accumulation (TNA) and potassium accumulation (TKA) were significantly positively correlated with total root length, root superficial area, and root volume. N application could alleviate the detrimental effects of shading on total nutrient accumulation and grain yield in rice.

Rice (*Oryza sativa L.*) is one of the most important food crops globally, being the primary food source for more than one-third of the world’s population^1^. In China, more than 60% of the population lives on rice, and rice makes up 40% of the country’s total grain production^2^. Therefore, the production of rice becomes more and more important because of the increase of the population of both China and the world^3^,^4^.

Nitrogen (N) is generally needed in most rice-producing environments, and its importance during the growth of this crop is well documented^5^,^6^. N fertilizer application considerations have been an effective production input which contributes significantly to rice yield improvement^7^,^8^. N topdressing application has been found to increase grain protein content and head yield in rice^9^,^10^,^11^. However, an excessive input of chemical fertilizer in rice production often leads to a series of environmental problems^12^,^13^,^14^. Thus a responsible nitrogen application becomes increasingly important for sustainable agronomic production^15^.

Light is one of the most critical environmental factors that determine proper rice growth and development. Ren *et al.*^16^ found that low light stress severely affected rice yield in the regions of Yunnan and Guizhou provinces. Sun *et al.*^17^ reported that photosynthesis rate of sword leaves decreased significantly when light irradiance was reduced to 40% of natural light irradiance during the heading stage in hybrid rice, resulting in a decrease in dry matter accumulation, and also an altered redistribution of photosynthetic products^18^,^19^. The study by Li *et al.*^20^ also demonstrated that dry matter accumulation and grain yield were decreased under shading treatments.

South China serves as one of the important staple rice cultivation regions, which can play a vital role during rice production in China. Root is the most important organ which can absorb water and inorganic nutrients. The growth of rice root can affect grain yield and qualities. However, there are few studies on the relationship among root morphologies, nitrogen accumulation, and physiological characteristics under shading conditions and different nitrogen treatments in different rice genotypes. The main objectives of this study were to: (1) evaluate the effects of shading under different N treatments on photosynthesis parameters, root morphological characteristics, total nutrient accumulation, and grain yield of rice; and (2) elucidate the relationship between total nutrient accumulation and root morphological characteristics under shading conditions and different nitrogen treatments.

## Results

### Grain yield and its components

There were some remarkable differences in both yield and yield components for the nitrogen and shading treatments in the three rice genotypes for both late season in 2011 and early season in 2012 (Tables 1 and 2). In the late season, grain yield under HN was significantly higher than that for MN and NN, which were 6.70, 5.56, and 5.38 t ha^−1^, respectively. There was no apparent difference in yield between MN and NN. Significant differences were also found in the number of valid panicles, spikelets per panicle, seed setting rate, and 1000-grain-weight under the different nitrogen treatments. There were marked increases in the number of valid panicles and spikelets per panicle under HN, which were 13.09 panicles hill^−1^ and 142.10 spikelets per panicle, respectively. However, significant decreases in seed setting rate and 1000-grain-weight were found under HN, compared with MN and NN, which were 69.43, 77.09, 79.29%, 22.49, 23.44, and 23.16 g, respectively. Grain yield under DS was significantly less than that of SS and NS, which were 4.77, 5.81, and 7.06 t ha^−1^, respectively. There also was a marked difference in yield between DS and SS.

Significant differences were also found in seed setting rate and 1000-grain-weight under the shading treatments. There were significant decreases in seed setting rate and 1000-grain-weight for DS compared with NS, which were 71.25, 82.29%, 22.64, and 23.31 g, respectively. As for different rice cultivars, there were significant differences in seed setting rate and 1000-grain-weight. The highest seed setting rate and 1000-grain-weight were found for *TY*, which were 76.50% and 23.48 g, respectively. Significant interaction effects were found between nitrogen treatments and rice cultivars for the number of spikelets per panicle, seed setting rate, and 1000-grain-weight. There were also significant interaction effects between nitrogen and shading treatment for the number of valid panicles, spikelets per panicle, seed setting rate, 1000-grain-weight, and yield. Also, significant interaction effects between rice cultivars and shading treatments were also found for seed setting rate and 1000-grain-weight.

The same trend happened for the early season in 2012 (Table 2). Grain yield under the HN treatment was significantly higher than that for MN and NN, which were 8.41, 7.34, and 6.56 t ha^−1^, respectively. There was also a significant difference in yield between MN and NN. Significant differences were also found in the number of valid panicles, seed setting rate, and 1000-grain-weight under the different nitrogen treatments. There was a marked increase in the number of valid panicles for HN, which was 10.52 panicle hill^−1^. However, a significant decrease in 1000-grain-weight was found for HN compared with NN, which were 21.97 and 22.98 g, respectively. Grain yield under DS was significant less than that for SS and NS, which were 6.64, 7.32, and 8.35 t ha^−1^, respectively. There was also a conspicuous difference in yield between DS and SS. Significant differences were also found in seed setting rate and 1000-grain-weight under the shading treatments. A significant decrease was observed in seed setting rate and 1000-grain-weight for DS compared with NS, which were 73.15, 87.82%, 22.09, and 22.92 g, respectively. Regarding rice cultivars, there were significant differences in the number of spikelets per panicle, seed setting rate, and 1000-grain-weight. The highest number of spikelets per panicle was found in *PZ*, which was 177.29. And the highest seed setting rate and 1000-grain-weight were for *YJ* and *TY,* respectively. Significant interaction effects also were found between nitrogen treatments and rice cultivars for number of spikelets per panicle and 1000-grain-weight. There were also significant interaction effects among rice cultivars and shading treatments for number of spikelets per panicle. And significant interaction effects between nitrogen and rice cultivars and shading treatments were also found for number of spikelets per panicle.

### Photosynthetic parameters

**Figure 1:**
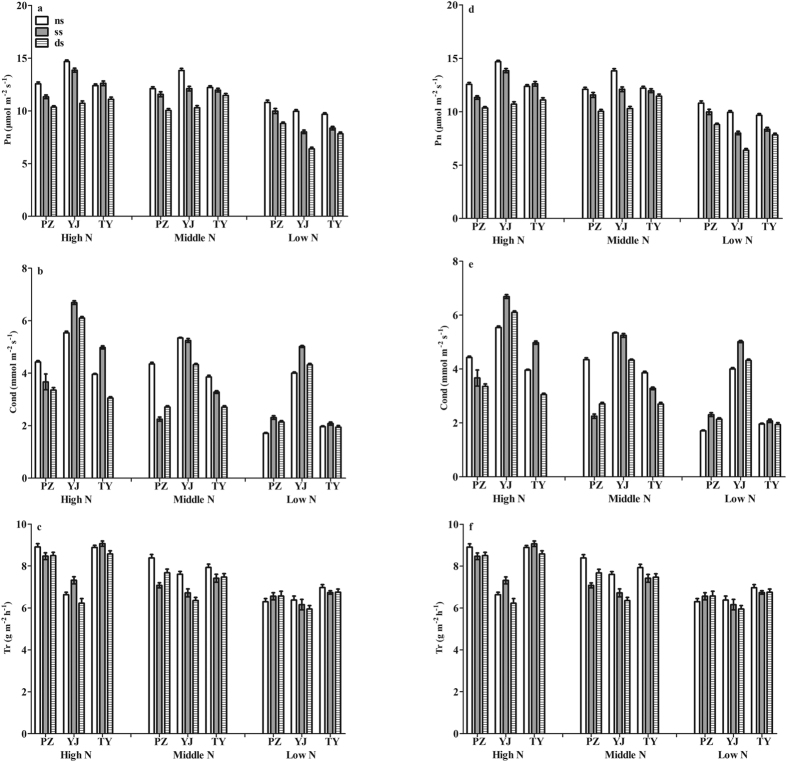
Effects of nitrogen and shading on photosynthesis parameters of sword leaves six days following treatment termination in the late season of 2011 and early season of 2012. Note: Photosynthesis rate (**a,d**), stomatal conductance (**b,e**), and transpiration rate (**c,f**) of sword leaves for 2011 and 2012, respectively.

In the late season, compared with NN, Pn and Cond in the sword leaves under MN and HN was significantly higher, which were 10.59, 10.34, 9.12 μmol m^−2^ s^−1^, 0.57, 0.54, and 0.43 mmol m^−2^ s^−1^, respectively. There was no significant difference in Pn and Cond between MN and HN. Significant differences were also found for the different shading treatments. Compared with the NS treatment, there were marked decreases in Pn and Cond under DS, which were 9.34, 0.42 mmol m^−2^ s^−1^, 10.57, and 0.62 mmol m^−2^ s^−1^, respectively. Regarding rice cultivars, there were significant differences in Pn and Cond. Pn and Cond of *YJ* were the largest; however, *PZ* was the smallest, which were 11.18, 8.97 μmol m^−2^ s^−1^, 0.71, and 0.39 mmol m^−2^ s^−1^, respectively. There were also significant interaction effects among nitrogen, shading, and rice cultivar treatments for Pn and Cond in the sword leaves. Significant effects of nitrogen and shading were also found in Tr. Compared to NN, there were significant increases for Tr under MN and HN, which were 5.11, 6.77, and 6.18 g m^−2^ h^−1^, respectively. Compared with NS, there was a significant decrease in Tr for SS and DS, which were 6.64, 5.32, and 6.11 g m^−2^ h^−1^, respectively. There also were significant differences for Tr among rice cultivars. Tr for YJ was the largest; however, PZ was the smallest, which were 7.40 and 5.27 g m^−2^ h^−1^, respectively. There were also significant interaction effects among nitrogen, shading, and rice cultivars for Tr in the sword leaves (Fig. 1a–c).

The same trend was observed for the early season in 2012 (Fig. 1d–f). Pn in the sword leaves for HN and MN was significantly higher than that for NN, which were 12.18, 11.73, and 8.86 μmol m^−2^ s^−1^, respectively. There was no significant difference in Pn between HN and MN. Significant differences were found for Pn under different shading treatments. Compared with NS, there was a significant decrease in Pn under DS and SS. Marked interaction effects on Pn among nitrogen, shading, and cultivar were found. Significant differences were also found for Cond under different nitrogen treatments. There was a significant increase in Cond with an increase in nitrogen application rate. The highest Cond was for HN, and the lowest was for NN. There was also a marked difference in Cond among cultivars. The highest Cond was for *YJ*; however, the lowest was for *PZ*. A significant difference was also found for intercellular CO_2_ concentration (Ci) between the two cultivars. Ci of *YJ* was significantly higher than that for *PZ* and *TY*. There was a significant difference in Tr for the different nitrogen treatments. Compared with NN, there was a significant increase in Tr for HN and MN. There was no significant difference between HN and MN, which were 7.56 and 7.28 g m^−2^ h^−1^, respectively. Significant interaction effects were found between nitrogen treatments and rice cultivars for the number of spikelets per panicle and 1000-grain-weight. There were also significant interaction effects among nitrogen, shading, and cultivars for Tr. Compared with NN, Pn and Cond of the sword leaves for MN and HN was significantly higher.

### Root morphological characteristics

For the late season, total root length, root superficial area, and root volume increased significantly for HN, compared with MN and NN. However, there was no significant difference for total root length, root superficial area, and root volume of rice between MN and NN. Significant differences were also found for total root length, root superficial area, and root volume under the shading treatments. The highest total root length, average root diameter, and root volume were for NS; however, the lowest was for DS, which were 13.48 × 10^3^, 12.81 × 10^3^ cm hill^−1^, 0.4944, 0.4608 mm, 25.09, and 23.54 cm^3^ hill^−1^, respectively (Table 3). There were also significant differences in total root length, root superficial area, and root volume between *TY* and *PZ.* The highest total root length, root superficial area, and root volume was for *TY;* however, the lowest was for *PZ,* which were 13.84 × 10^3^, 11.69 × 10^3^ cm hill^−1^, 2076.58, 1785.13 cm^2^ hill^−1^, 25.75, and 22.36 cm^3^ hill^−1^, respectively. However, the highest average root diameter was found for *YJ,* and the lowest was found for *TY*, which were 0.4891 and 0.4683 mm, respectively. There were also significant interaction effects between nitrogen application and rice cultivar for average root diameter and root volume. Significant interaction effects between nitrogen and shading treatments were also found for total root length, root superficial area, average root diameter, and root volume. And there were significant interaction effects among nitrogen application, rice cultivar, and shading treatment for root superficial area and root volume.

Significant effects on root morphological characteristics for nitrogen application and shading treatments were also observed for the early season (Table 4). Total root length, root superficial area, and root volume increased significantly for HN, compared with MN and NN. However, there was no significant difference in total root length, root superficial area, and root volume between MN and NN. Significant differences in total root length, root superficial area, average root diameter, and root volume were also found for the shading treatments. The highest total root length, average root diameter, and root volume were for NS; however, the lowest was observed for DS, which were 14.11 × 10^3^, 11.73 × 10^3^ cm hill^−1^, 0.5028, 0.4774 mm, 25.58, and 22.98 cm^3^ hill^−1^, respectively. There was a marked difference in total root length, root superficial area, and root volume among the rice cultivars. The highest total root length was for *TY,* and the lowest was for *PZ. T*he lowest average diameter was found for *TY,* and the highest for *PZ.* There were also significant interaction effects between nitrogen application and shading treatments for total root length, root superficial area, and root volume.

### Total nitrogen accumulation (TNA), phosphorus accumulation (TPA), and potassium accumulation (TKA)

It was observed that both nitrogen application and shading treatments affected significantly TNA, TPA, and TKA for the three rice genotypes, in both late and early seasons (Tables 5 and 6). For the late season, TNA, TPA, and TKA increased significantly under HN, compared with NN. However, there was no significant difference for TNA, TPA, and TKA, compared with MN and NN. Significant differences were also found for TNA, TPA, and TKA under the shading treatments. Highest TNA and TPA were for DS; however, the highest TKA was for NS. There were also significant differences in TNA and TKA between *TY* and *PZ*. The highest TNA and TKA were for *TY* and the lowest was for *PZ.* There were also significant interaction effects between nitrogen application and rice cultivar for TPA. Significant interaction effects between nitrogen and shading treatments were also found for TPA and TKA. There were significant interaction effects among nitrogen applications, rice cultivars, and shading treatments for TPA and TKA.

There were significant effects for TNA, TPA, and TKA under nitrogen application and shading treatment for the early season (Table 6). TNA and TPA increased significantly under HN, compared with NN. However, there was no significant difference in TNA between HN and MN. Significant differences in TNA and TPA were also found for the shading treatments. The highest TPA was under DS; however, the highest TNA was observed for NS. There was no marked difference in TKA of rice for the shading treatments. Significant differences were also found in TNA and TKA among the cultivars. The highest TPA and TKA were for *YJ*, and the lowest was for *PZ.* There were also significant interaction effects among nitrogen application, cultivar, and shading treatments for TPA.

### Correlations between root morphological characteristics and nutrient absorption

TKA and TNA showed a nearly consistent relationship with root morphological characteristics (Table 7). TKA had significant and positive correlations with total root length and root volume at the 5% probability level. And there also was a significantly positive correlation between TKA and total root superficial area at the 1% probability level. TNA also had remarkably positive correlations with total root length at the 5% probability level. Furthermore, the correlation among TNA and total root superficial and root volume was also markedly positive at the 1% probability level. Both TKA and TNA showed a consistent relationship for average root diameter, although the positive correlation was not significant at the 5% probability level. There were also positive correlations between TPA and root morphological characteristics (total root length, root superficial area, average root diameter, and root volume), although the correlation coefficients were not significant at the 5% probability level. The data revealed that nutrient absorption by rice plants was determined primarily by total root length, root superficial area, and root volume under both nitrogen and shading treatments for both late and early seasons.

## Discussion

Appropriate N management can significantly increase both net photosynthesis rate and yield of rice^7^,^21^. Shading treatment during the mid-tillering or heading stages can markedly decrease photosynthesis rate in rice leaves, which leads to less soluble carbohydrate available for transport to the grain of rice^22^. Our results showed that Pn and Cond in the sword leaves under MN and HN were significantly higher than that under NN. Tr in the sword leaves under MN was markedly higher than that under NN. An increase of N application could increase glutamine synthetase and nitrate reductase activities in sword leaves (data no shown), improve the nitrogen content in rice leaves, thus leading to an increase of photosynthetic ability. Compared with NS, there were marked decreases in Pn and Cond under DS. The likely cause was a decrease in the content of superoxide dismutase, and an increase of malonaldehyde in the leaves under the double-shading treatment, which resulted in a weakened photosynthetic ability^22^. There were also significant interactive effects among N, shading, and rice cultivars on Pn and Cond in the sword leaves. Significant effects of N and shading were also found in Tr. Regarding different rice cultivars, the highest Pn, Cond, and Tr was for *YJ*, and the lowest was for *PZ*.

The morphological characteristics of a plant root system can significantly influence the uptake of water and nutrients. Root morphological characteristics can be affected by fertilizer application and light irradiance^16^,^23^,^24^. Mandal *et al.*^25^ found that integrated use of mineral fertilizers and farmyard or green manure could markedly improve crop root length density, root volume, and root dry weight, as well as the depth of root penetration. Yang *et al.*^26^ also reported that incorporation of organic manure into paddy soil could improve root morphological characteristics and root activity of rice plants by increasing root density, active absorption area, and root surface phosphatase activity. The present study showed that total root length, root superficial area, and root volume increased remarkably under HN. Significant decreases were also found in total root length, root superficial area, and root volume under DS. Our previous study showed that this may be associated with the improvement of photosynthetic rate of rice leaves under the N application and no shading treatments^27^, which can lead to more carbohydrate being available for translocation to the root, which was in agreement with the results found by Liu *et al.*^28^ and Li *et al.*^20^. Regarding cultivar differences, *TY* demonstrated the highest total root length, root superficial area, and root volume, and *PZ* was the lowest.

Reasonable N fertilizer application can increase TNA and improve nitrogen use efficiency in rice^2^,^4^,^15^,^29^. Qiao *et al.*^30^ emphasized that applying nitrogen also improved phosphorus use efficiency in rice. Kyi *et al.*^31^ also reported that improving nitrogen use efficiency could increase TPA and TKA in rice. The results in the present study showed that TNA, TPA, and TKA increased markedly under HN. Significant differences were also found in TNA, TPA, and TKA under the shading treatments. The highest TNA and TPA were under DS; however, the highest TKA was for the NS treatment. Optimum uptake of nitrogen, as a primary nutrient, is a key requirement for the rice crop to be able to accomplish high uptake rates of P and K. Rice plants experiencing high nitrogen use efficiency can produce more leaves, show vigorous growth of shoots and root, and thus absorb more P and K^7^,^21^. And significant interactive effects between nitrogen and shading treatments were also found for both TPA and TKA of rice in the present study. When all data from both nitrogen rates and the shading treatments were pooled, there was a positive correlation among TNA/TKA, total root length, root superficial area, and root volume. These results demonstrated that strong root systems could increase the absorption of TNA and TKA in rice (Table 7).

N fertilization plays a key role in the production of rice. Reasonable nitrogen management can not only increase grain yield of rice, but also improve nitrogen recovery efficiency^10^,^11^,^32^, which alleviates environmental pollution^14^. The present study showed that there was significantly higher grain yield under HN than under MN and NN. Also, a large increase in the number of valid panicles and spikelets per panicle under HN was observed. However, there was a significant decrease in seed-setting rate and 1000-grain-weight under HN, which is not in agreement with the results found by Deng *et al.*^33^ and Liu *et al.*^34^. In the present study, the effects of shading on crop yield varied in different rice cultivar, which was not only in relation to rice cultivar tolerance to low light stress, but also to light character, shading duration, crop growth period, and shading degree^17^,^35^. Further studies are needed to clarify these observations under various crop growing conditions.

Many studies have shown a reduction in yield caused by shading (i.e., reduced light) stress^36^,^37^. It has been reported that the decrease in grain yield was caused by a decrease in the number of valid panicles per m^2^ and 1000-grain-weight^38^,^39^. There was no significant decrease in the number of valid panicles per m^2^ and spikelets per panicle (Table 1 and 2). The significant decrease in seed setting rate and 1000-grain-weight were the main reasons for the decline in grain yield. Shading delayed plant flowering^40^, hindered pollen germination, and increased the number of degenerated spikelets^41^. Thus, the number of unfilled spikelets increased, and there was an apparent reduction in spikelet filling^42^. Zhang *et al.*^37^ reported that 1000-grain-weight was largely determined by photosynthate distribution after heading. The present study observed that there was a significant decrease in seed setting rate, 1000-grain-weight, and yield under DS. Significant interactive effects were found between nitrogen and shading treatments on the number of valid panicles, spikelets per panicle, seed setting rate, 1000-grain-weight, and yield. Regarding the three rice cultivars evaluated, the higher seed setting rate and 1000-grain-weight was for *Tianyou998*. Our results also showed that N application could alleviate the detrimental effects of shading on the number of productive tillers and grain yield in rice.

In this study, the main aims are mainly about the effects of nitrogen and shading on root morphology, nitrogen accumulation, and photosynthetic parameters in different rice genotypes, further researches are to analyze the physiological and anatomical reasons of the differences in different nitrogen fertilizer rate, shading conditions, and rice genotypes. In order to mitigate the negative influences of severe shading, it is necessary that breeding and planting more rice varieties with high tolerance to low light conditions. And adopting optimum agronomic measurements such as silicon or organic fertilizer application to cope with low light stress is also beneficial.

## Methods

### Experimental design and cultural practices

Field experiments were conducted during the late season (July-November) in 2011 and the early season (March-July) in 2012 in two adjacent fields at the College of Agriculture’s Experimental Farm, South China Agricultural University (SCAU), Guangzhou, Guangdong Province, China (113.18′E, 23.10′N, elevation 18 m). The mean monthly air temperature, mean daily radiation, precipitation, and average humidity during the rice growing season are shown in Table 8. The paddy soil had 23.24 g kg^−1^ organic C, 1.14 g kg^−1^ total N, 1.14 g kg^−1^ total P, 24.41 g kg^−1^ total K, 61.34 mg kg^−1^ available P, and 127.04 mg kg^−1^ available K.

Treatments were arranged in a split–split plot design, with nitrogen rate as the main plot, rice cultivar as the subplot, and shading as the sub-subplot. Sub-subplot size was 20 m^2^. All the treatments had four replications. Main plot treatments were three nitrogen rates (0, 120, and 180 kg N ha^−1^, and written as NN, MN, and HN, respectively). Subplot treatments were three rice cultivars, which were *Peizataifeng*, *Tianyou998* and *Yuejingsimiao2*, and written as *PZ*, *TY* and *YJ*, respectively. *Peizataifeng* (SCAU), (*Peiai64S* × *taifengzhan*), a two-line hybrid rice type, was developed by plant space breeding by the College of Agriculture, South China Agricultural University. Its entire growth period is about 125 and 115 days for early and late planting, respectively. *Tianyou998 (Tianfeng A* × *Guanghui 998*), a three-line hybrid rice type, was developed by the Rice Institute, Guangdong Academy of Agricultural and Science. Its entire growth period is about 123 and 117 days for early and late planting, respectively. *Yuejingsimiao2* (the inbred rice type) was developed by the Rice Institute, Guangdong Academy of Agricultural and Science. Its entire growth period is about 120 and 125 days for early and late planting, respectively. Sub-subplot treatments consisted of three shading treatments, which were covered with a single layer of shade netting (single shading, SS), another with two layers of shade netting (double shading, DS), and the third left uncovered to create a light gradient (no shading, NS). Their transmittance rates were measured with a light meter (Model LI-250; LI-COR, USA). The shading treatments were achieved using enclosures covered with black polypropylene netting, reducing the light intensity to 33.2 and 11.5% attenuation, respectively. The netting was draped over a 6.5 m × 5 m × 4 m wooden frame to ensure adequate ventilation, and fully covered the respective shaded plots. The shading treatments were initiated at the first heading stage, and continued for 10 days. Nitrogen in the form of urea was split-applied at basal (BS), mid-tillering (MT), and panicle initiation (PI). The N- splitting pattern for both seasons was 40% (BS) + 30% (MT) + 30% (PI). P fertilizer in the form of single superphosphate (SSP) was applied at the rate of 120 kg ha^−1^ as P_2_O_5_ (basal). Potassium (potassium chloride) at 180 kg K_2_O ha^−1^ was applied with a split of 60% (basal) and 40% at the panicle initiation stage (PI).

Fifteen-day-old seedlings from wet-bed nurseries were transplanted at the rate of 2 seedlings per hill at a spacing of 20.0 cm × 20.0 cm (2.5 × 10^5^ hills ha^−1^) on Aug 7 and April 20, respectively. Each fertilizer-treatment plot was surrounded by a 35-cm wide ridge which was covered with plastic film. The plastic film was installed to a depth of 20 cm below the soil surface three days before transplanting. All crop managements were in accordance with standard cultural practices. The plots were flooded three days after transplanting, and a water depth of 4–10 cm was maintained until seven days before maturity, at which time the field was drained. Standard chemical products were used to manage diseases, insects, and weeds.

### Sampling and measurements

Ten days following shading treatment termination, 30 hills of plants from each plot were collected for calculation of average panicle number per hill. Five representative hills of the plants then were separately sampled and divided into leaf blades, stems plus sheathes, and grain. The samples were oven-dried at 80 °C (to constant weight), weighed, then milled, and stored dry until analyzed for total nitrogen concentration. Nitrogen, Phosphorus, and potassium concentrations in each plant part were determined according to Lithourgidis *et al.*^43^. Nitrogen, Phosphorus, and potassium uptake, and also accumulation in the aboveground tissues were calculated^44^.

### Photosynthetic parameters

Net photosynthetic rate (Pn), stomatal conductance (Cond), and transpiration rate (Tr) of the sword leaves in the ten days shading treatment (following termination) were determined with a LI-6400XT Portable Photosynthesis System (LI-COR, Inc., USA) in both 2011 and 2012. The measurements were conducted using the traditional open system. The PAR was set at 1200 μmol m^−2^s^−1^, which was provided by a 6400-2B LED light source. An average value was calculated from five sword leaves from each replicate.

### Roots sampling and measurements

Root sampling measurements were accomplished using a modification of the protocol described by Steingrobe *et al.*^45^. Following preparation of the paddy field, eight mesh bags (25–30 cm) were put into the buried cylinder (25–30 cm) to a depth of 25 cm in each plot, then filled with uniform slurry. Two rice seedlings were transplanted into the mesh bag, assuring that the rice root would not come out, and that water and nutrients could enter at the transplanting stage. Finally, the buried cylinders were taken out of the soil. Eight mesh bags containing two rice seedlings each were taken out when the shading treatments were finished in the ten days, then all root rinsed carefully with clean tap water. The cleaned rice roots were taken to the lab for measurement of certain morphological characteristics (including total root length, average root diameter, root superficial area, and root volume), using a root analysis instrument WinRhizo-LA1600 (Regeng Instuments Inc., Quebec, Canada).

### Yield and its components

Grain yield and its components were measured according to the methods described by Peng *et al.*^46^.

### Data analysis

Data for each season were analyzed using the standard analysis of variance procedure (SAS Institute, 2003). Relationships among total nitrogen accumulation, total phosphorus accumulation, total potassium accumulation, and root morphological characteristics were evaluated using correlation analyses (Statistix, 2003). Means among treatments were compared based on the least significant difference test (LSD) at the 0.05 probability level.

## Additional Information

**How to cite this article**: Pan, S. *et al.* Effects of Nitrogen and Shading on Root Morphologies, Nutrient Accumulation, and Photosynthetic Parameters in Different Rice Genotypes. *Sci. Rep.* **6**, 32148; doi: 10.1038/srep32148 (2016).

## Figures and Tables

**Table 1:** Effects of nitrogen and shading on yield and its components in different rice genotypes for the late season of 2011.

Treatments			Productive panicle per hill	Spikelet per Panicle	Seed-setting rate(%)	1000-grain -weight(g)	Harvested yield (t ha^−1^)
HN	PZ	NS	13.00	143.46	64.82	21.24	7.97
		SS	13.23	156.27	60.14	21.60	6.83
		DS	11.63	157.94	59.71	20.13	5.50
	YJ	NS	13.77	137.45	81.35	21.60	7.37
		SS	13.47	124.77	63.42	21.63	6.75
		DS	11.70	124.02	56.89	20.85	5.69
	TY	NS	14.28	153.62	83.86	26.17	8.50
		SS	13.43	125.48	74.92	25.69	6.53
		DS	13.27	155.90	79.77	23.46	5.14
		**mean**	**13.09 a**	**142.10 a**	**69.43 b**	**22.49 b**	**6.70 a**
MN	PZ	NS	11.57	119.18	88.94	26.44	7.54
		SS	12.40	108.88	72.42	24.93	6.27
		DS	12.73	119.64	84.75	24.32	4.03
	YJ	NS	13.83	113.73	79.23	22.42	6.47
		SS	12.50	131.83	64.64	21.66	5.40
		DS	11.73	131.15	56.83	23.30	4.07
	TY	NS	13.27	129.43	89.70	22.15	6.38
		SS	12.80	118.06	69.38	23.20	5.07
		DS	12.70	114.54	87.95	22.53	4.85
		**mean**	**12.61 ab**	**122.19 b**	**77.09 a**	**23.44 a**	**5.56 b**
NN	PZ	NS	11.90	118.43	86.86	21.69	6.75
		SS	11.73	128.31	79.84	22.14	5.22
		DS	10.17	126.98	81.29	21.39	4.60
	YJ	NS	11.10	145.93	94.33	25.96	6.47
		SS	11.30	106.53	90.12	25.07	5.82
		DS	10.70	114.67	78.24	24.01	5.15
	TY	NS	10.43	123.89	76.88	22.15	6.12
		SS	10.97	128.13	70.25	22.21	4.43
		DS	11.40	122.71	55.79	23.78	3.88
		**mean**	**11.08 b**	**121.21 b**	**79.29 a**	**23.16 a**	**5.38 b**
Analysis of Variance		year*year	ns	ns	ns	ns	ns
		f	**	**	**	*	*
		v	ns	ns	*	*	ns
		shade	*	ns	**	**	**
		f*v	ns	*	*	*	ns
		f*shade	**	*	*	*	*
		v*shade	ns	ns	*	**	ns
		f*v*shade	ns	ns	*	*	ns

Within a column, means followed by the same letter are not significantly different at the 0.05 probability level according to least significant different test (LSD0.05). ns means not significant different according to LSD0.05; *means significant different according to LSD0.05. **means significant different according to LSD0.01; f means fertilizer; v means variety; the same as below.

**Table 2:** Effects of nitrogen and shading on yield and its components in different rice genotypes for the early season of 2012.

Treatments			Productive panicle per hill	Spikelet per Panicle	Seed-setting rate(%)	1000-grain -weight(g)	Harvested yield (t ha^−1^)
HN	PZ	NS	9.67	169.79	81.53	20.49	8.43
		SS	9.53	157.72	74.27	19.87	8.10
		DS	8.82	201.03	66.29	19.76	6.95
	YJ	NS	10.72	126.09	94.34	22.42	9.22
		SS	10.88	146.30	86.85	21.49	7.87
		DS	11.88	167.31	71.79	20.41	8.02
	TY	NS	10.97	152.14	87.54	24.74	10.30
		SS	12.03	142.18	79.22	24.34	7.93
		DS	10.20	139.84	73.03	24.23	8.85
		**mean**	**10.52 a**	**155.82 a**	**79.43 ab**	**21.97 b**	**8.41 a**
MN	PZ	NS	10.08	206.60	79.61	20.87	8.13
		SS	10.05	190.12	77.74	21.20	7.83
		DS	10.25	194.69	67.36	20.12	7.04
	YJ	NS	9.95	121.53	90.11	22.35	7.35
		SS	9.78	148.87	76.21	21.15	7.13
		DS	9.98	131.55	74.27	20.65	5.14
	TY	NS	10.95	148.94	91.49	25.68	8.61
		SS	10.32	128.12	69.89	24.61	7.80
		DS	10.05	163.70	71.00	24.36	7.05
		**mean**	**10.16 a**	**159.35 a**	**77.52 b**	**22.33 a**	**7.34 b**
NN	PZ	NS	7.78	169.35	85.29	21.81	8.28
		SS	9.05	168.13	79.71	21.71	6.15
		DS	9.62	138.17	70.16	22.52	5.53
	YJ	NS	9.23	152.61	88.48	21.80	7.25
		SS	8.42	148.04	83.90	21.37	6.66
		DS	8.72	165.25	75.49	21.08	5.86
	TY	NS	8.30	143.70	91.94	26.09	7.62
		SS	8.43	184.10	80.18	24.73	6.41
		DS	8.72	135.62	88.98	25.67	5.33
		**mean**	**8.70 b**	**156.11 a**	**82.68 a**	**22.98 a**	**6.56 c**
Analysis of Variance		year*year	ns	ns	ns	ns	ns
		f	**	ns	*	**	**
		v	ns	*	*	*	ns
		shade	ns	ns	*	*	*
		f*v	*	*	ns	**	ns
		f*shade	ns	ns	ns	ns	ns
		v*shade	ns	*	ns	ns	ns
		f*v*shade	ns	*	ns	ns	ns

**Table 3:** Effects of nitrogen and shading on root morphological characteristics in different rice genotypes for the late season of 2011.

Treatments			Total Root Length (×10^3^ cm hill^−1^)	Superficial Root Area (cm^2^ hill^−1^)	Average Root Diameter (mm)	Total Root volume (cm^3^ hill^−1^)
HN	PZ	NS	13.87	2024.21	0.4725	23.82
		SS	12.24	2018.16	0.4668	27.30
		DS	11.71	1792.52	0.4452	22.13
	YJ	NS	15.97	2332.42	0.5313	27.86
		SS	13.88	2168.97	0.5056	27.52
		DS	12.81	2002.71	0.4862	25.71
	TY	NS	17.21	2362.52	0.4939	26.68
		SS	14.05	2300.26	0.4935	30.90
		DS	12.32	1905.91	0.4854	24.00
		**mean**	**13.79 a**	**2100.85 a**	**0.4867 a**	**26.21 a**
MN	PZ	NS	12.58	1989.81	0.5196	26.08
		SS	9.71	1511.25	0.5022	19.32
		DS	11.57	1607.11	0.5173	18.08
	YJ	NS	13.24	2086.98	0.5381	27.07
		SS	10.24	1639.19	0.5151	22.19
		DS	15.76	2053.77	0.4220	21.70
	TY	NS	12.05	2001.14	0.4339	27.29
		SS	10.95	1805.10	0.4291	24.31
		DS	15.90	2097.78	0.4093	22.53
		**mean**	**12.45 b**	**1865.79 b**	**0.4763 a**	**23.17 b**
NN	PZ	NS	11.78	1817.29	0.4790	22.84
		SS	11.30	1558.61	0.4700	17.70
		DS	10.48	1747.19	0.4710	23.93
	YJ	NS	12.30	1794.51	0.4847	21.11
		SS	11.31	1690.05	0.4518	20.69
		DS	11.89	1870.38	0.4667	24.62
	TY	NS	12.31	1861.30	0.4963	23.08
		SS	16.86	2231.73	0.4931	23.85
		DS	12.89	2123.49	0.4802	29.14
		**mean**	**12.35 b**	**1854.95 b**	**0.4770 a**	**23.00 b**
Analysis of Variance		year*year	ns	ns	ns	ns
		f	*	*	ns	*
		v	**	*	*	*
		shade	*	ns	*	*
		f*v	ns	ns	**	*
		f*shade	*	*	*	*
		v*shade	ns	*	ns	ns
		f*v*shade	ns	*	ns	*

**Table 4:** Effects of nitrogen and shading on root morphological characteristics in different rice genotypes for the early season of 2012.

Treatments			Total Root Length (×10^3^ cm hill^−1^)	Superficial Root Area (cm^2^ hill^−1^)	Average Root Diameter (mm)	Total Root volume (cm^3^ hill^−1^)
HN	PZ	NS	15641.85	2400.98	0.5007	30.06
		SS	14554.20	2279.64	0.5153	29.45
		DS	13090.40	2000.74	0.4749	23.85
	YJ	NS	14964.85	2636.91	0.5223	34.35
		SS	14492.00	2622.36	0.5056	34.16
		DS	12565.09	2263.42	0.5053	29.22
	TY	NS	18279.55	2500.18	0.4675	28.16
		SS	16393.49	2380.29	0.4668	28.32
		DS	13656.36	1998.26	0.4550	22.65
		**mean**	**14848.64 a**	**2342.53 a**	**0.4904 a**	**28.91 a**
MN	PZ	NS	11326.86	1806.04	0.5446	24.33
		SS	10445.73	1429.97	0.5151	19.79
		DS	9788.53	1684.23	0.4856	23.63
	YJ	NS	13281.78	1904.96	0.5225	24.65
		SS	11243.55	1781.87	0.5196	23.44
		DS	12771.17	2069.59	0.5077	26.70
	TY	NS	18877.78	2463.09	0.4220	26.03
		SS	14568.44	1738.19	0.4291	19.25
		DS	12570.26	1442.35	0.4339	17.70
		**mean**	**12763.79 b**	**1813.37 b**	**0.4867 a**	**22.83 b**
NN	PZ	NS	10068.09	1656.23	0.5229	20.62
		SS	12131.80	1652.38	0.4919	22.18
		DS	9721.18	1441.90	0.4710	17.23
	YJ	NS	12134.87	1891.42	0.5449	24.19
		SS	13295.76	2228.22	0.5432	24.81
		DS	11464.71	1925.23	0.4963	27.69
	TY	NS	12398.43	1519.37	0.4777	17.79
		SS	15970.94	2121.11	0.4518	22.75
		DS	9942.67	1485.14	0.4673	18.17
		**mean**	**11903.16 b**	**1769.00 b**	**0.4963 a**	**21.72 b**
Analysis of Variance		year*year	ns	ns	ns	ns
		f	*	*	ns	*
		v	*	*	*	*
		shade	*	*	*	*
		f*v	ns	ns	ns	ns
		f*shade	*	*	ns	*
		v*shade	ns	ns	ns	ns
		f*v*shade	ns	ns	ns	ns

**Table 5:** Effects of nitrogen and shading on total nitrogen accumulation, phosphorus accumulation, and potassium accumulation in rice genotypes for the late season of 2011.

Treatments			TNA (kg ha^−1^)	TPA (kg ha^−1^)	TKA (kg ha^−1^)
HN	PZ	NS	44.72	25.49	245.31
		SS	62.91	29.98	197.48
		DS	81.21	38.91	186.68
	YJ	NS	72.15	33.71	280.82
		SS	90.42	43.80	292.33
		DS	81.80	50.70	208.65
	TY	NS	89.87	32.73	307.51
		SS	101.84	35.59	241.95
		DS	100.36	46.80	191.04
		**mean**	**80.59 a**	**37.52 a**	**239.08 a**
MN	PZ	NS	59.25	37.50	170.13
		SS	55.06	33.76	240.70
		DS	48.46	35.80	143.33
	YJ	NS	67.14	32.35	261.37
		SS	58.56	31.20	211.03
		DS	54.59	25.19	155.84
	TY	NS	64.04	23.52	193.93
		SS	56.38	24.98	221.06
		DS	65.81	33.19	228.11
		**mean**	**58.81 b**	**30.83 b**	**202.83 ab**
NN	PZ	NS	40.62	8.29	160.05
		SS	34.93	12.07	155.31
		DS	57.05	17.69	173.68
	YJ	NS	55.57	12.90	195.28
		SS	52.05	19.21	233.99
		DS	63.03	14.89	198.37
	TY	NS	47.16	9.05	211.73
		SS	57.61	12.83	204.63
		DS	57.71	12.72	203.76
		**mean**	**51.75 b**	**13.29 c**	**192.98 b**
Analysis of Variance		year*year	ns	ns	ns
		f	*	*	*
		v	*	ns	*
		shade	*	*	*
		f*v	ns	*	ns
		f*shade	ns	*	*
		v*shade	ns	*	ns
		f*v*shade	ns	*	*

**Table 6:** Effects of nitrogen and shading on total nitrogen accumulation, phosphorus accumulation, and potassium accumulation in rice genotypes for the early season of 2012.

Treatments			TNA (kg ha^−1^)	TPA (kg ha^−1^)	TKA (kg ha^−1^)
HN	PZ	NS	63.04	24.72	147.75
		SS	60.08	37.07	122.41
		DS	52.20	40.83	120.41
	YJ	NS	70.02	33.19	198.74
		SS	71.13	44.60	187.67
		DS	61.68	54.66	164.71
	TY	NS	67.08	16.46	113.12
		SS	61.89	19.54	155.95
		DS	46.10	24.09	111.58
		**mean**	**61.47 a**	**32.80 a**	**146.93 b**
MN	PZ	NS	73.46	14.54	132.13
		SS	64.68	21.64	173.17
		DS	55.76	19.74	133.21
	YJ	NS	79.18	47.86	173.22
		SS	78.49	39.88	179.19
		DS	52.57	42.50	187.65
	TY	NS	64.79	24.21	143.71
		SS	68.00	41.62	166.32
		DS	70.80	24.95	161.59
		**mean**	**67.53 a**	**30.77 a**	**161.13 a**
NN	PZ	NS	58.81	19.87	136.95
		SS	41.54	17.89	115.44
		DS	43.61	27.54	141.56
	YJ	NS	62.80	11.31	189.67
		SS	50.98	27.47	146.63
		35.32	20.53	129.06	
	TY	NS	45.63	13.61	124.74
		SS	43.33	22.45	121.60
		DS	41.20	18.34	133.31
		**mean**	**47.02 b**	**19.89 c**	**137.66 b**
Analysis of Variance		year*year	ns	ns	ns
		f	*	**	**
		v	ns	**	*
		shade	*	*	ns
		f*v	ns	ns	ns
		f*shade	ns	ns	ns
		v*shade	ns	ns	ns
		f*v*shade	ns	*	ns

**Table 7:** Correlation coefficients between root morphology and nutrient absorption in 2011 and 2012 Data were the averages from all the plots in both late and early seasons.

Parameter	TPA	TKA	TNA
Total root length	0.0154	0.4313*	0.4385*
Total superficial area	0.0481	0.5547**	0.5551**
Average root diameter	0.0515	0.1393	0.1036
Total root volume	0.0860	0.4856*	0.5939**

**Table 8:** Mean monthly air temperature, mean daily radiation, precipitation, and average humidity during the rice growing season in 2011 and 2012.

Time	Temperature (°C)	Solar radiation (MJm^−2^ d^−1^)	Precipitation (mm)	Average humidity (%)
2011				
July	29.10	13.95	189.70	78.00
August	30.10	19.00	43.00	70.00
September	27.70	12.08	175.80	72.00
October	23.60	10.49	199.30	73.00
November	21.90	12.87	111.20	71.00
2012				
March	18.30	5.53	28.70	80.00
April	23.60	6.63	340.50	83.00
May	27.40	11.03	269.70	80.00
June	28.20	9.85	198.50	79.00
July	28.90	16.29	279.60	77.00
