# Corrigendum: Effects of Nitrogen and Shading on Root Morphologies, Nutrient Accumulation, and Photosynthetic Parameters in Different Rice Genotypes

**DOI:** 10.1038/srep45611

**Published:** 2017-03-30

**Authors:** Shenggang Pan, Haidong Liu, Zhaowen Mo, Bob Patterson, Meiyang Duan, Hua Tian, Shuijin Hu, Xiangru Tang

Scientific Reports 6: Article number: 3214810.1038/srep32148; published online: 08 25 2016; updated: 03 30 2017

The original version of this Article contained a typographical error in the spelling of the author Shuijin Hu, which was incorrectly given as Shuijing Hu. This has now been corrected in the PDF and HTML versions of the Article.

